# Empathy Development in Preschoolers With/Without Hearing Loss and Its Associations with Social-Emotional Functioning

**DOI:** 10.1007/s10802-024-01271-0

**Published:** 2024-12-09

**Authors:** Zijian Li, Boya Li, Yung-Ting Tsou, Johan H. M. Frijns, Qi Meng, Shannon Yuen, Liyan Wang, Wei Liang, Carolien Rieffe

**Affiliations:** 1 Unit of Developmental and Educational Psychology, Institute of Psychology, Faculty of Social and Behavioral Sciences, Leiden University, Leiden, The Netherlands; 2 Otorhinolaryngology and Head and Neck Surgery, Leiden University Medical Center, Leiden, The Netherlands; 3 Leiden Institute for Brain and Cognition, Leiden, The Netherlands; 4 China Rehabilitation Research Center for Hearing and Speech Impairment, Beijing, China; 5 Department of Human Media Interaction, Faculty of Electrical Engineering, Mathematics and Computer Science, University of Twente, Enschede, The Netherlands; 6 Department of Psychology and Human Development, Institute of Education, University College London, London, UK

**Keywords:** Empathy, Development trajectory, Preschoolers, Social-emotional functioning

## Abstract

Empathy plays a crucial role in children’s social-emotional development. There is an increasing trend in recent studies to recognize empathy as a multi-dimensional construct, consisting of three distinct hierarchical levels: emotion contagion, attention to others’ feelings and prosocial behaviors (Hoffman, *Motiv Emot*, 14(2), 151–172, 1990). The present study is amongst the first to use a longitudinal approach to examine the development trajectories of the distinct empathic levels, based on a sample of Chinese preschoolers aged 2 to 6 years, half of the sample being deaf or hard-of-hearing (DHH). Our results showed that according to the parental observation, DHH preschoolers manifested similar extent of emotion contagion and attention to others’ feelings as their TH (typically hearing) peers over preschool years. Yet, DHH preschoolers showed fewer prosocial behaviors, compared to their TH peers. As for the longitudinal associations over time, emotion contagion contributed to more internalizing and externalizing behaviors in both groups; whilst attention to others’ feelings contributed to fewer internalizing behaviors in only DHH children. Prosocial behaviors contributed to better social competence, and fewer internalizing and externalizing behaviors in both DHH and TH children just as expected. These outcomes imply that the early intervention or special education may be useful to safeguard children’s empathic development, shrinking the gaps between DHH and TH children; but meanwhile, cultural factors might cause latent effects on children’s understandings of empathy and impact on how empathy “regulates” children’s social-emotional functioning, in a Chinese cultural context.

## Introduction

Empathy, the ability to vicariously experience and understand others’ emotions and to alleviate their emotional stress, is essential for navigating social interactions as well as fostering interpersonal bonds (e.g., Decety & Jackson, 2006; Hoffman, 1990; Rieffe et al., 2010). It is also crucial for children’s early social-emotional development. Higher levels of empathy are associated with better social competence, and fewer internalizing and externalizing behaviors (e.g., Neumann et al., 2016; Noten et al., 2019; Tully & Donohue, 2017). However, the development of empathy among deaf or hard-of-hearing (DHH) children may pose more challenges compared to typically hearing (TH) children, as they encounter additional obstacles in accessing their social environment when raised in predominantly hearing environments (Morgan et al., 2014; Dirks et al., 2020). For instance, when loud background noises are present during conversations, DHH children may receive only partial or distorted information (Calderon & Greenberg, 2011; Rieffe et al., 2015). The problems of missing out on social learning opportunities cannot be solved completely with hearing-aid (HA) devices or a cochlear implant (CI), which work best in one-to-one interactions/communications in relatively quiet environments (Caldwell & Nittrouer, 2013; Misurelli & Litovsky, 2015). These restrictions on social participation that DHH children often experience may have a profound negative impact on their emotional socialization. DHH children are reported to show lower levels of sharing and understanding emotions, including empathy (e.g., Moeller, 2007; Netten et al., 2015; Rieffe et al., 2015). To date, only a few studies have explored the empathic development of young DHH children, and notably, they operationalized empathy as a general, unidimensional concept (e.g., Dirks et al., 2017; Peterson, 2016). However, increasing numbers of recent studies indicated empathy to be multi-dimensional in both adults and children (e.g., Da Silva et al., 2022; Hojat et al., 2018; Rieffe et al., 2010). Considering the importance of understanding early developmental stages in hearing and DHH children to ensure timely intervention, the present study aimed to explore how different components of empathy develop over the preschool years among DHH and TH children, and how these empathic components affect children’s social-emotional development.

Hoffman (1990) proposed a multi-level model of empathy where three distinct levels develop sequentially in the early years of life. “Emotion Contagion”, also known as “affective empathy”, is the first and basic level of empathy, referring to the extent to which a person can be affected by others’ emotional expressions or behaviors. Newborn infants can already be affected by the emotional expressions of others: their attempts to mirror/mimic others’ verbal statements, facial expressions, or body gestures can already be observed even since their first year of life, which presumably triggers similar arousal in themselves (Bernhardt & Singer, 2012). Therefore, when one infant bursts out crying in a room, other nearby infants may follow spontaneously. Prior research showed that this capacity, which is supposed to be innate (De Waal, 2008), is observable in both TH and DHH preschoolers. According to parental reports, DHH preschoolers using CIs manifested similar levels of emotion contagion as their hearing peers (Ketelaar et al., 2015; Tsou et al., 2021).

Although emotion contagion was related to more prosocial behaviors in DHH and TH preschool children (Ashori & Aghaziarati, 2023; Da Silva et al., 2022; Fink & de Rosnay, 2023), too much emotion contagion can result in high personal distress and internalizing behaviors (e.g., Geng et al., 2012; Rieffe et al., 2010; Tsou et al., 2021). Possibly, empathizing with others’ (negative) emotions can be overwhelming for young children who have not yet developed sufficient emotion regulation abilities (Rieffe et al., 2010). As children grow older and become more and more capable of regulating their own emotional arousal, emotion contagion tends to decrease slightly, which was observed during the preschool years in both DHH and TH children (Tsou et al., 2021). This implies that emotion contagion is largely innate and is influenced little by hearing loss.

The second level of empathy, “Attention to Others’ Feelings”, emerges around the second year of life (Hoffman, 1990). “Attention to Others’ Feelings” represents the ability of children to suppress their own idiosyncratic desires and switch their focus of attention to others’ needs and feelings, which is a developmental marker of children’s socialization process. From a developmental perspective, as daily environments are full of information and emotional stimuli, toddlers develop a basic sense of self-awareness at around the age of one that allows them to distinguish others’ emotional arousal from their own feelings. Upon realizing that their contagious arousal is triggered by others’ emotions, children could be less overwhelmed by contagious emotions and temporarily suppress their own desires, helping them to shift attention toward the other person in need (Rieffe et al., 2010). Such a shift of attention from “self-focused” to “other-focused” relies on input from their social environment, and prepares children for further social interactions, such as caring about others’ desires/needs and performing prosocial behaviors.

For DHH children, it may be more difficult to be aware of the emotions of others when their attention is not directed to the source. Yet, even when they do shift their attention to others’ emotional displays, DHH children may still struggle to understand the situation and to interpret verbal feedback about their social behaviors (Ketelaar et al., 2015). During communication with peers, DHH children may often take in partial or distorted information (e.g., Calderon & Greenberg, 2011). These obstacles may limit DHH children’s opportunities of social/incidental learning, making it more challenging for them to develop an understanding of others’ emotions and perspectives (Morgan et al., 2014). Longitudinal research showed similar levels of attention to others’ feelings in DHH preschoolers and their TH peers, but these DHH children manifested a higher increase over time (Tsou et al., 2021).

Notably, while attention to others’ feelings is related to better social competence in DHH and TH preschoolers (Ashori & Aghaziarati, 2023; Bandstra et al., 2011; Da Silva et al., 2022; Li et al., 2023; Rieffe et al., 2010), DHH and TH preschool children who showed larger/faster increases in their attention over time were at greater risks of exhibiting internalizing behaviors (Tsou et al., 2021). Presumably, focusing attention on others’ negative emotions increases young children’s personal distress who do not yet have sufficient emotion regulation abilities (Eisenberg, 2006).

“Prosocial behaviors” form the third level of empathy, emerging as children’s abilities to feel, understand, and respond to others’ emotions increase. The initiation of prosocial acts may need understanding of others’ intentions, desires, and beliefs, however, preschool children do not yet understand the intrinsic causes of others’ emotional expressions due to their still-developing cognitive abilities (Broekhof et al., 2015). Yet, despite not fully understanding others’ perspectives and mental states, preschoolers still manifest the motives and action tendencies to alleviate the influence of negative feelings in others, and such altruistic motives often take the form of concrete behaviors, such as helping, sharing, or comforting (Beeler-Duden et al., 2022; Zahn-Waxler et al., 1992). As a matter of fact, spontaneous prosocial behaviors are already observable in preschoolers, and the quality and quantity of these behaviors improve as their emotion regulation and perspective-taking abilities develop (Flook et al., 2019; Tsou et al., 2021). Furthermore, higher levels of prosocial behaviors may be related to better-developed social competence and fewer internalizing and externalizing behaviors among TH preschoolers (Caputi et al., 2012; Da Silva et al., 2022; Rieffe et al., 2010; Salerni & Caprin, 2022).

DHH children manifest lower prosocial motives and fewer prosocial behaviors than their TH peers do (e.g., Netten et al., 2015; Tsou et al., 2021). Although the group differences persist during the preschool years, both TH and DHH children were found to display increasing levels of prosocial behaviors during the period (Netten et al., 2015; Rieffe et al., 2010; Takamatsu et al., 2021; Tsou et al., 2021). These findings suggest that although DHH children feel and attend to others’ emotions, they do not demonstrate prosocial actions towards distressed others to the same extent as their TH peers do. These findings can be attributed to their difficulties in understanding complex social situations and social knowledge to support them to react in these situations, as well as a lack of empowerment to take action (Tsou et al., 2021). Considering the essential role of prosocial acts in facilitating various kinds of interpersonal relationships, these group differences observed in prior studies might account for the lower social competence and higher frequencies of behavioral problems of DHH preschoolers (e.g., Chao et al., 2015; Netten et al., 2015). Cross-sectional research also found that prosocial behaviors were related to better social competence and fewer behavioral problems in DHH preschoolers (Ashori & Aghaziarati, 2023). Yet, longitudinal research did not find a significant effect of prosocial behaviors on internalizing and externalizing behavioral problems (Tsou et al., 2021).

### Present Study

To the best of our knowledge, only one research examined the development trajectory of empathy in DHH preschoolers and how distinct empathic components differentially affect children’s psychosocial development (Tsou et al., 2021). To further extend our knowledge in this area, the present study aimed to use a longitudinal design with 2-time points to investigate the development of empathy and its associations with social-emotional functioning in Chinese DHH and TH preschoolers.

Our first goal was to compare the different empathic levels between DHH and TH preschoolers. Based on prior studies, we expected the DHH group to show similar levels of emotion contagion and attention to others’ feelings, whereas lower levels of prosocial behaviors, compared to their TH peers (Ketelaar et al., 2013, 2015; Tsou et al., 2021).

The second goal was to explore the development of empathy in preschool years among DHH and TH preschoolers. We expected emotion contagion to decrease whereas prosocial behaviors to increase among both DHH and TH preschoolers at a similar pace (Tsou et al., 2021). Moreover, we expected the DHH preschoolers to show a significant increase in attention to others’ feelings over time, whilst the TH preschoolers may have a slower increase over time in this regard (Tsou et al., 2021).

Our third goal was to investigate the longitudinal effects of these three empathic levels on DHH/TH preschoolers’ social-emotional functioning, i.e., social competence, internalizing, and externalizing behaviors. According to previous research, we expected that all three empathic levels would be related to better social competence over time in both groups (e.g., Da Silva et al., 2022; Simon & Nader-Grosbois, 2023). Moreover, we expected emotion contagion and attention to others’ feelings to be related to more internalizing behaviors over time (Rieffe et al., 2010; Tsou et al., 2021); and prosocial behaviors to fewer internalizing and externalizing behaviors, over time in both TH and DHH preschoolers (Ashori & Aghaziarati, 2023).

## Method

### Participants

This study is part of a larger-scaled longitudinal research project investigating the early development of social-emotional functioning in Chinese DHH and TH children (e.g., Yuen et al., 2022).

A total of 250 children (DHH: 129; TH: 121) aged 21 to 84 months (M = 48.61 months, SD = 12.39 months at the first wave) participated in the study. Two waves of data were collected on this sample, with an average interval of 14.62 (SD = 4.33) months. The participants were recruited from the China Rehabilitation Research Center for Hearing and Speech Impairment (CRRCHSI) in China, from 2019 to 2020. Most of the DHH participants had severe hearing loss with mean unaided hearing thresholds of 78 dB at the better-hearing ear, and 87 dB at the worse-hearing ear. 86% of the DHH participants were using a hearing aid (HA) or a cochlear implant (CI) (average hearing thresholds after adjustments: 23 dB at the better hearing ear; 33 dB at the worse hearing ear).

CRRCHSI is a research and intervention institution located in Beijing, China, dedicated to providing early interventions and support to children with hearing loss. CRRCHSI has an affiliated kindergarten offering preschool education to both DHH and TH children. All DHH children receive special education for two years before being integrated into mixed classes with TH peers. Their special education and intervention programs are designed to support DHH preschoolers’ language and social development. Spoken language is the emphasis in education, and sign language is not preferred to be used in their daily routines. The demographic characteristics of our DHH participants are similar to their TH peers (Table 1). No group difference was found when comparing the group means.

**Table 1:** The personal characteristics of participants

	DHH (*n* = 129)	TH (*n* = 121)
*Personal characteristics*		
Age at time 1, years, mean (*SD*)	47.20 (14.95)	50.12 (8.70)
Gender, *n* (%)		
Male	74 (57.4%)	64 (52.9%)
Female	55 (42.6%)	57 (47.1%)
Non-verbal intelligence score, mean (*SD*)	100.23 (16.10)	103.56 (12.25)
*Socioeconomic status*,* mean (SD)*		
Maternal education^a^	3.64 (0.93)	4.04 (0.99)
Paternal education^a^	3.62 (0.83)	4.07 (0.90)
Annual household income^b^	4.11 (0.85)	3.76 (1.99)
*Hearing characteristics*		
Age of identification, months, mean (*SD*)	14.78 (14.87)	
Hearing device, *n* (%)		
Unilateral CI	5 (3.93%)	
Unilateral HA	1 (0.79%)	
Bilateral CI	15 (11.8%)	
Bilateral HA	24 (18.9%)	
Bilateral CI + HA	54 (42.5%)	
Not using any equipment	19 (15.0%)	
Missing data	9 (7.09%)	
HA use, months, mean (*SD*)	33.95 (16.45)	
CI use, months, mean (*SD*)	29.09 (12.84)	
Unaided hearing threshold, better ear, mean (SD)^c^	82.73 (25.11)	
Unaided hearing threshold, worse ear, mean (SD)^c^	87.63 (29.21)	
Aided hearing threshold, better ear, mean (SD)^c^	30.20 (7.81)	
Aided hearing threshold, worse ear, mean (*SD*)^c^	40.29 (16.90)	

*Note*: SD = standard deviation; HA = hearing aid; CI = cochlear implant

^a^Mean (SD) of the values representing different conditions, the values: 1 = “Primary school & below”, 2 = “Junior high”, 3 = “High school”, 4 = “University or College”, 5 = “Postgraduate & above”

^b^Mean (SD) of the values representing different conditions, the values: 1 = “<€3,000”; 2 = “€3,000-€15,000”; 3 = “€15,000-€20,000”; 4 = “€20,000-€25,000”; 5 = “€25,000-€40,000”; 6 = “€40,000-€65,000”; 7 = “€65,000-€130,000”; 8 = “> €130,000”

^c^These values represent the hearing threshold of the worse/better ear (decibels; dB)

** *p* < 0.001, *p < 0.05 between DHH and TH children

Our recruitment inclusion criteria were: (1) for DHH children, they should have a pre-lingual hearing loss (hearing loss prior to three years of age) with a minimum threshold of 40 decibels in the better-hearing ear (calculated as averaging their unaided hearing thresholds at 500, 1000, 2000, 4000 Hz); (2) for both groups, they should have a non-verbal IQ of 80 at the minimum, with no other psychiatric diagnoses.

The non-verbal IQ data of the participants were retrieved from CRRCHSI. The Griffiths Mental Development Scales (Griffiths & Huntley, 1996) were used to assess the non-verbal intelligence of the participants. The testing and scoring were performed by the researchers from CRRCHSI, prior to this study.

### Procedure

Prior to data collection, the research protocol was approved by CRRCHSI and the ethics committee of Leiden University. The teachers of the participating preschoolers were contacted prior to data collection. We informed the teachers about the requirements of this study and then acquired their agreement to comply with these requirements.

The purposes, execution, data management, privacy policy of the study, and the voluntary nature of participation were stated in the informed consent. The caregivers of the children were requested to sign the informed consent, prior to the data collection. Upon receiving the signed informed consent, the teachers distributed the (paper-form) questionnaires to the children. These questionnaires were taken home by the children to their caregivers and were brought back to the teachers after completion. The teachers finally collected all the responses and sent them to the researchers. In the meantime distributing the paper questionnaires, a link to the portal with an online questionnaire was sent (via email) to the participants who could not access the paper questionnaires, allowing them to fill out the questionnaire online.

### Measures

#### Empathy

To assess the empathic levels of preschool children, the Chinese version of the Empathy Questionnaire (EmQue) was used. The EmQue is a parent report designed to measure the manifestations of empathy among preschoolers, originally in Dutch (Rieffe et al., 2010), and validated in many different languages such as Italian (Grazzani et al., 2017), Spanish (Lucas-Molina et al., 2018), Portuguese (Da Silva et al., 2022), and Japanese (Takamatsu et al., 2021), showing robust psychometric properties. The EmQue was also validated in Chinese and the three-level empathy construct applied to Chinese preschool children as well (Li et al., 2024a). The EmQue consists of 19 items, each item depicts a specific behavior in a specific situation, divided over three scales that measure the three distinct levels of empathy. More specifically, the “Emotional Contagion” scale (6 items) assesses to what extent children are affected by their peers’ emotions (e.g., “When another child cries, my child gets upset too”). The “Attention to Others’ Feelings” scale (7 items) measures to what extent children’s attention is attracted by others’ expressions (e.g., “My child looks up when another child cries”). The “Prosocial Behavior” scale (6 items) evaluates how motivated children are to help others in various occasions (e.g., “When two children are quarrelling, my child tries to stop them”). Accordingly, the three scales focus on different aspects/levels of empathy. Respondents are instructed to rate to which extent each item’s depiction represented the child’s behavior in the past two months upon a 3-point scale (0 = never, 1 = sometimes, 2 = often). A higher score indicates a higher disposition of the corresponding empathic feature. Respondents are encouraged to rate all items, even if some items do not apply to them. Table 2 shows the descriptive statistics of the three scales, and the McDonald’s omegas ranged from 0.78 to 0.86.

**Table 2:** The characteristics and reliabilities of study variables at each time point

	NO. items	Scale	McDonald’s omega	DHH		TH	
				Mean	SD	Mean	SD
*Time 1*							
Emotional contagion	6	0–2	0.81	0.54	0.35	0.54	0.37
Attention to others	7	0–2	0.78	1.32	0.36	1.34	0.37
Prosocial behaviors	6	0–2	0.84	0.90	0.43	0.96	0.42
Social competence	10	0–2	0.81	1.30	0.33	1.43	0.32
Internalizing behaviors	25	0–3	0.89	0.75	0.32	0.84	0.13
Externalizing behaviors	18	0–3	0.90	0.82	0.38	0.86	0.39
*Time 2*							
Emotional contagion	6	0–2	0.85	0.53	0.37	0.59	0.40
Attention to others	7	0–2	0.80	1.36	0.35	1.37	0.36
Prosocial behaviors	6	0–2	0.86	1.10	0.42	1.13	0.36
Social competence	10	0–2	0.73	1.47	0.27	1.50	0.28
Internalizing behaviors	25	0–3	0.91	0.88	0.14	0.85	0.13
Externalizing behaviors	18	0–3	0.90	0.78	0.35	0.77	0.36

#### Internalizing and Externalizing Behaviors

To assess the severity of internalizing and externalizing behaviors, the Early Childhood Inventory − 4th edition (ECI-4, Sprafkin et al., 2002) parent checklist was used. Given that there was no validated Chinese version of ECI-4, we used a (back-)translation procedure to convert the English version of ECI-4 into Chinese (see “Translation procedure” below). The ECI-4 consists of 9 scales and 108 items, which screen for 15 social-emotional and behavioral disorders. Respondents are instructed to rate on a 4-point scale (0 = never, 1 = sometimes, 2 = often, 3 = very often) to what extent their child manifested each behavior in the past three months. A higher score indicates a higher likelihood of manifesting the corresponding symptom. Following Ketelaar et al. (2017)’s method, we combined several (sub)scales to measure internalizing and externalizing behaviors:

To measure “Internalizing Behaviors”, four ECI-4 scales were combined, which included the “Major Depressive Disorder” scale (10 items, e.g., “The child cries, freezes, or avoids communicating with others when they are placed in an uncomfortable social setting”); the “Separation Anxiety” scale (8 items, e.g., “Having nightmares upon being separated from their parents”); the “Social Phobia” scale (3 items, e.g., “Climbs to the parents’ bed at the middle of the night”); the “Generalized Anxiety” scale (4 items, e.g., “Is overly afraid of/avoiding some particular objects or situations”).

As for “Externalizing Behaviors”, two scales were combined for the assessment, including the “Oppositional Defiant Disorder” scale (8 items, e.g., “Refuses to do what you asked him/her to”), and the “Conduct Disorder” scale (10 items, e.g., “Intentionally fights with others”).

#### Social Competence

To assess social competence, the Chinese version of the Strengths and Difficulties Questionnaire (SDQ; Lai et al., 2010) was used. The SDQ consists of 5 scales, each of which has 5 items, that screen for children’s social and emotional functioning. Respondents are instructed to rate to what extent their child manifested each symptom/behavior on a 3-point scale (0 = not true, 1 = somewhat true, 2 = certainly true), based on observations of their child’s performances in the past six months.

Following the method of Ketelaar et al. (2017), we combined two scales of SDQ to measure “Social Competence”, including the “Peer Problems” scale (5 items, e.g., “Is relatively lonely and playing with himself or herself”), and the “Prosocial Behavior” scale (5 items, e.g. “Is very willing to share with other children their candies, toys, and pens, etc.”).

Table 2 reports the descriptive statistics of the measurements used in this study. McDonald’s omegas ranged from 0.78 to 0.91, indicating that the internal consistencies of the used measurements are sufficient.

Notably, although two items of “Social Competence” may conceptually overlap with the Prosocial Behaviors scale of the EmQue, the examination to see if the data met the assumption of collinearity suggested that multi-collinearity between the two indices was not a concern (Tolerance = 1.00, VIF = 1.00).

### Translation Procedure

As the ECI-4 has not been translated or validated in Chinese, we used a back-translation method to adapt it to Chinese (Brislin et al., 1973). The first translation was performed by a senior psychologist from our research team who was fluent in English and Chinese. Thereafter, a back-translation from Chinese to English was conducted by another senior psychologist who was bilingual. The translations were checked in terms of consistency. The inconsistencies in translation were resolved by discussions with our research team.

### Statistical Analyses

Linear mixed models (LMMs) were used to analyze our data. Our longitudinal data had two waves (time points) that were nested within participants. The stepwise method was used to enter variables into the models. The criterion used for evaluating the LMM models was: lower − 2 Log likelihood [− 2LL] values in likelihood ratio tests (Wood et al., 2008). The best-fitting model was determined by selecting the one with the lowest − 2LL. When two models showed equal − 2LL values, the simpler model was preferred over the more complex one. Unstandardized (B) and standardized (Beta) estimates were both reported. When B and Beta values indicated different results, greater consideration was given to the Beta values as they are considered more reliable in revealing the effect sizes of predictors (Lorah, 2018).

To investigate the development of empathy during preschool years in DHH and TH children, we began with three baseline unconditional-means models including only fixed and random intercepts for the three empathic levels (emotion contagion, attention to others’ feelings, and prosocial behaviors), respectively. Next, age was added to the models as a fixed factor, to evaluate how these empathic levels changed across two time points. Thereafter, group as a fixed factor was entered into the models, to evaluate if there were group differences regarding the reported levels of empathy. The interaction of age with group was entered into the models at last, to assess if the developmental trajectories varied between groups.

To examine the contribution of the three empathic levels to predicting the social-emotional development of DHH and TH preschoolers, we began with three models with only fixed and random intercepts of social competence, internalizing, and externalizing behaviors, respectively. Firstly, mean and change variables were created for each of the empathic levels. The values of the mean variables were the mean scores of time 1 and time 2. The values of the change variables were computed by subtracting participants’ scores of time 1 from those of time 2. Specifically, the mean variables were used to assess how the mean levels of empathy were related to social-emotional development (between-subject effects), and the change variables were used to assess how an increase or decrease of empathy was related to the social-emotional development (within-subject effects). After creating the mean and change variables, we started to construct the models. Age, group, and their interaction were entered into the baseline model in the first step. Next, we added the mean and change variables of each empathic level to the corresponding model. Lastly, we entered the interactions of group with mean/change variables to the models, to examine whether there were group differences for the predictions.

All the analyses mentioned above were conducted using R version 4.2.3 (R Core Team, 2021). Linear mixed models (LMMs) were performed using the “lme4” package (Bates et al., 2015). Figures were crafted using the package “ggplot2” (Wickham, 2016).

### Missing Data Analysis

240 participants filled in all the questionnaires at time 1. Due to the Covid-19 pandemic, the researchers lost contact with some of these participants during the second collection. Caregivers of 128 participants who attended the first wave collection provided data again for the second wave collection. The attrition rate of our longitudinal data is thus 48.8%. According to simulation research, attribution rates of lower than 50% would be considered acceptable, as the estimations based on which data were not largely biased compared to data with no attrition (e.g., Gustavson et al., 2012). Considering also that LMMs are relatively robust in handling data with attrition, we consider the attrition rate of 48.8% suitable for LMM analyses (Pan & Zhan, 2020).

Little’s MCAR was used to assess whether our missing values were distributed randomly. At time 1, less than 3% missing values were found, and they were missing completely at random: χ2 = 3923.35, df = 5643, *p* > 0.05. At time 2, less than 0.5% of data were missing, which was missing at random: χ2 = 3658.62, df = 5643, *p* > 0.05.

## Results

### The Development of Empathy During Preschool Years

**Fig. 1:**
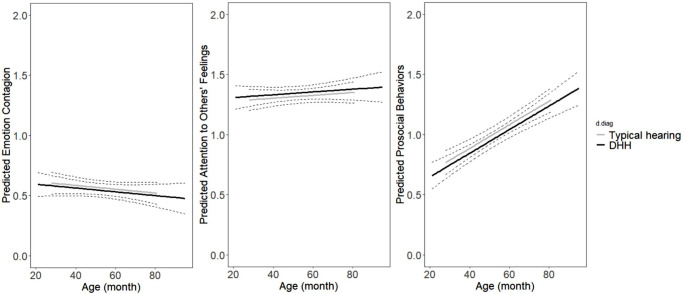
Longitudinal graphic representation of the predicted values based on the optimal fitting models of empathy. *Note*: Black solid lines represent the predicted mean values of DHH children, while Grey solid lines represent the predicted mean values of TH children. Dotted lines represent the upper and lower bounds of the 95% confidence interval

Table 3 shows the best-fitting LMM models for the development of emotion contagion, attention to others’ feelings, and prosocial behaviors. Figure 1 depicts the development trajectories and group differences of the three empathic levels.

**Table 3:** Fixed and random effects of the best models in predicting the empathic levels over time

	Emotion contagion -2LL = 293.1		Attention to others’ feelings -2LL = 274.7		Prosocial behaviors -2LL = 355.2			
	B (SE)	CI [low, high]	B (SE)	CI [low, high]	B (SE)	CI [low, high]	Beta (SE)	CI [low, high]
*Fixed effects*								
Intercept	**0.55 (0.02)**	[0.51, 0.59]	**1.33 (0.02)**	[1.29, 1.38]	**0.47 (0.08)**	[0.31, 0.63]	**1.00 (0.02)**	[0.95, 1.04]
Age (linear)	**-**	-	**-**	-	**0.009 (0.001)**	[0.006, 0.012]	**0.28 (0.04)**	[0.19, 0.36]
Group	-	-	**-**	-	**-**	-	**-**	-
*Random effects*								
Residual	**0.08 (0.29)**	[0.25, 0.32]	**0.08 (0.28)**	[0.25, 0.31]	**0.09 (0.30)**	[0.26, 0.34]	**0.09 (0.30)**	[0.26, 0.34]
Intercept	**0.05 (0.23)**	[0.18, 0.28]	**0.05 (0.22)**	[0.17, 0.27]	**0.08 (0.27)**	[0.22, 0.32]	**0.08 (0.27)**	[0.22, 0.32]

*Note*: B = Unstandardized estimates of fixed effects; Beta = Standardized Estimates of fixed effects; SE = standard error. CI [low, high] = lower to upper bounds, of the 95% confidence interval. Significant fixed/random effects are marked as using bolded font

For emotion contagion and attention to others’ feelings, the best-fitting model (having the lowest − 2LL) includes only a fixed and random intercept. Adding the effects of age and group did not improve the models. This suggests that emotion contagion and attention to others’ feelings stayed unchanged with age in TH and DHH preschoolers. Also, TH and DHH preschoolers manifested a similar extent of emotion contagion and attention to others’ feelings across the preschool years.

As for prosocial behaviors, the best-fitting model included a fixed effect of age (b = 0.009, beta = 0.28, t = 6.65, *p* < 0.001, 95% CI: [0.19, 0.36]). The result indicates that prosocial behaviors increased with age among TH and DHH preschoolers. Adding the effect of the group did not further improve the model, suggesting no group differences between TH and DHH preschoolers.

### Longitudinal Associations Between Empathy and Social-Emotional Functions

Table 4 shows the best-fitting models for predicting the contribution of empathy to the development of social-emotional development in DHH and TH children.

**Table 4:** Fixed and random effects of the best predicting models of empathy contributing to social-emotional functioning over time

	Social competence -2LL = 71.4				Internalizing behaviors -2LL = -132.9				Externalizing behaviors -2LL = 251.6			
	B (SE)	CI [low, high]	Beta (SE)	CI [low, high]	B (SE)	CI [low, high]	Beta (SE)	CI [low, high]	B (SE)	CI [low, high]	Beta (SE)	CI [low, high]
*Fixed effects*												
Intercept	**0.69 (0.09)**	[0.51, 0.86]	**1.40 (0.02)**	[1.37, 1.43]	**0.85 (0.06)**	[0.82, 0.96]	**0.83 (0.01)**	[0.81, 0.85]	**1.13 (0.10)**	[0.94, 1.33]	**0.82 (0.02)**	[0.77, 0.86]
Age (linear)	**0.005 (0.001)**	[0.003, 0.007]	**0.15 (0.03)**	[0.09, 0.21]	0.001 (0.001)	[-0.001, 0.002]	0.02 (0.02)	[-0.03, 0.07]	**-0.004 (0.001)**	[-0.007, -0.001]	**-0.12 (0.04)**	[-0.20, -0.04]
Group	**-0.08 (0.03)**	[-0.14, -0.02]	**-0.08 (0.03)**	[-0.14, -0.02]	**-0.09 (0.02)**	[-0.14, -0.04]	**-0.03 (0.02)**	[-0.07, -0.01]	0.03 (0.04)	[-0.05, 0.12]	0.03 (0.04)	[-0.05, 0.12]
Emotion contagion - mean	**-**	-	-	-	**0.10 (0.04)**	[0.03, 0.17]	**0.07 (0.02)**	[0.02, 0.11]	**0.17 (0.07)**	[0.02, 0.30]	**0.11 (0.05)**	[0.02, 0.21]
Attention to others’ feelings - mean	**0.10 (0.05)**	[0.004, 0.205]	**0.07 (0.03)**	[0.002, 0.136]	**-**	-	**-**	-	**-**	-	**-**	-
Prosocial behaviors - mean	**0.34 (0.04)**	[0.25, 0.43]	**0.26 (0.03)**	[0.19, 0.33]	**-0.10 (0.03)**	[-0.15, -0.03]	**-0.08 (0.02)**	[-0.12, -0.02]	**-0.17 (0.06)**	[-0.29, -0.05]	**-0.13 (0.05)**	[-0.22, -0.04]
Emotion contagion - change	**-**	-	**-**	-	**0.06 (0.03)**	[0.004, 0.12]	**0.06 (0.03)**	[0.003, 0.12]	**0.13 (0.05)**	[0.04, 0.22]	**0.13 (0.05)**	[0.04, 0.22]
Attention to others’ feelings - change	**-**	-	-	-	-0.03 (0.03)	[-0.16, -0.04]	**-0.12 (0.03)**	[-0.18, -0.06]	**-**	-	**-**	-
Prosocial behaviors - change	**-**	-	-	-	-	-	-	-	-	-	-	-
Group * attention to others’ feelings - change	-	-	-	-	**-0.10 (0.03)**	[-0.15, -0.04]	**-0.15 (0.04)**	[-0.23, -0.06]	**-**	-	**-**	-
*Random effects*												
Residual	**0.05 (0.22)**	[0.20, 0.25]	**0.05 (0.22)**	[0.20, 0.25]	**0.04 (0.20)**	[0.19, 0.22]	**0.04 (0.20)**	[0.19, 0.22]	**0.06 (0.24)**	[0.21, 0.27]	**0.06 (0.24)**	[0.21, 0.28]
Intercept	**0.03 (0.16)**	[0.11, 0.20]	**0.03 (0.16)**	[0.11, 0.20]	**0.001 (0.001)**	[0.001, 0.005]	**0.001 (0.001)**	[0.001, 0.005]	**0.07 (0.27)**	[0.22, 0.31]	**0.07 (0.27)**	[0.22, 0.31]

*Note*: B = Unstandardized estimates of fixed effects; Beta = Standardized Estimates of fixed effects; SE = standard error. CI [low, high] = lower to upper bounds, of the 95% confidence interval. Significant fixed/random effects are marked as using bolded font

For social competence, the best-fitting model that had the lowest − 2LL included: fixed effects of age (b = 0.005, beta = 0.15, t = 5.15, *p* < 0.001), group (b = -0.08, beta = -0.08, t = -2.54, *p* = 0.012), the mean score of attention to others’ feelings (b = 0.10, beta = 0.07, t = 2.04, *p* = 0.042), and the mean score of prosocial behaviors (b = 0.34, beta = 0.26, t = 7.57, *p* < 0.001). The results meant that social competence increased with age, whilst DHH preschoolers manifested lower social competence than their TH peers. For both DHH and TH preschoolers, higher attention to others’ feelings, or more prosocial behaviors, was related to better-developed social competence.

For internalizing behaviors, the best-fitting model included the effects of group (b = -0.09, beta = -0.03, t = -3.70, *p* < 0.001), the mean and change scores of emotion contagion (mean score: b = 0.10, beta = 0.07, t = 2.82, *p* = 0.005; change score: b = 0.06, beta = 0.06, t = 2.82, *p* = 0.04), the mean score of prosocial behaviors (b = -0.10, beta = -0.08, t = -3.16, *p* = 0.001), the change score of attention to others’ feelings (b = -0.03, beta = -0.12, t = -1.15, *p* = 0.250), and the interaction between group and the change score of attention to others’ feelings (b = -0.10, beta = -0.15, t = -3.38, *p* < 0.001). The results indicated that internalizing behaviors did not change with age, whilst DHH preschoolers showed more internalizing behaviors than their TH peers. For both groups, higher levels or an increase over time in emotion contagion, as well as fewer prosocial behaviors, were predictive of developing/displaying more internalizing behaviors. Also, only in DHH preschool children, an increase over time in attention to others’ feelings was related to fewer internalizing behaviors (post-hoc analysis within the DHH group: b = -0.14, beta = -0.22, t = -4.18, *p* < 0.001).

For externalizing behaviors, the best-fitting model included effects of age (b = -0.004, beta = -0.12, t = -2.99, *p* = 0.003), the mean and change scores of emotion contagion (mean score: b = 0.17, beta = 0.11, t = 2.29, *p* = 0.023; change score: b = 0.13, beta = 0.13, t = 2.75, *p* = 0.006), and the mean score of prosocial behaviors (b = -0.17, beta = -0.13, t = -2.80, *p* = 0.006). The results suggested that preschoolers’ externalizing behaviors decreased with age, and TH and DHH preschoolers manifested similar levels of externalizing behaviors. For both groups, higher levels or an increase over time in emotion contagion, as well as fewer prosocial behaviors, were predictive of developing more externalizing behaviors.

## Discussion

Empathy is crucial to children’s social-emotional development. However, little research focused on the development of empathic components in DHH preschoolers. The present study is amongst the first to investigate the developmental trajectories of three empathic levels, i.e., emotion contagion, attention to others’ feelings, and prosocial behaviors, in DHH and TH preschool children, and how the empathic development is related to their psychosocial functioning. Unlike prior studies that found some differences in empathy between DHH and TH children (e.g., Ketelaar et al., 2015; Tsou et al., 2021), our study revealed similar levels and developmental trajectories between DHH and TH children regarding the three empathy components. In both groups, emotion contagion stayed stable over the preschool years, contributing to more internalizing behaviors as expected, whereas the expected relation with social competence was absent. Attention to others’ emotions also remained stable over time and was associated with better social competence in both DHH and TH children, whilst unexpectedly, an increase in attention to others’ feelings was related to fewer internalizing behaviors in DHH children only. Prosocial behaviors increased over time, contributing to better social competence and fewer internalizing and externalizing behaviors in both TH and DHH children, which was in line with prior findings. Below, we discuss these outcomes in greater detail.

Regarding emotion contagion, we had hypothesized a decrease in both TH and DHH children over time, based on the expectation that preschoolers would become less aroused by others’ emotions with their improved emotion regulation skills. Our study however found stable levels of emotion contagion over time similarly in the two groups. This finding may partly be explained by the age of this sample (mean age of 49 months at Time 1) and the time span of the study (mean interval of 15 months). For children at this age, they have already developed the ability to recognize that the personal distress they experience is the consequence of witnessing other people’s emotions, rather than from themselves. This ability to differentiate their own and other people’s emotions can help them better regulate their emotions, keeping those emotions at a certain level that is easier to manage while leaving them the mental space to pay attention and respond to other people in distress (Hoffman, 1990; Rieffe et al., 2010). With age, their skills for this process could still improve, leading to further decreases in emotion contagion (e.g., Dennis & Kelemen, 2009; Tsou et al., 2021), yet with the time span used in this study, we may fail to capture that.

Moreover, for children who had difficulties regulating their emotion contagion levels, thus manifesting overall higher levels of contagion or an increase over time in their contagion level, our study showed that these children were at greater risks of developing not only internalizing behaviors but also externalizing behaviors. Although most prior studies using the same empathy questionnaire found no relationship between contagion and externalizing behaviors (Da Silva et al., 2022; Li et al., 2024a), also one other study, including Dutch children with a CI, found a similar relationship over time (Tsou et al., 2021). These outcomes seem to converge with the explanation that the tendency to experience overarousal can motivate an individual to feel overwhelmed and to focus excessively on their own emotions in emotionally charged situations that further trigger defensive behaviors. Alternatively, cultural variances may have a role. Cross-cultural research showed that East-Asian individuals are usually more inter-dependent on each other in social contexts compared to Western individuals, hence they might be more easily and strongly affected when observing others’ negative emotional states (Atkins et al., 2016; Markus & Kitayama, 1991; Matsumoto et al., 2008).

Regarding attention to others’ emotions, in line with the literature, children who paid more attention to others’ feelings showed better social competence over time (Tsou et al., 2021). However, contradictory to Tsou and colleagues, our results indicated that an increase in attention to others’ feelings was associated with fewer, instead of more, internalizing behaviors in DHH preschoolers, whilst being unrelated to externalizing behaviors in both groups. Therefore, children’s attention to others’ feelings in our study seems a more positive factor, especially for the DHH population. Whilst overall the level of attention to others’ feelings was shown to be stable over time in our sample, it seems that DHH children who started with lower levels can benefit from increasing levels of attention towards others over time. An increase in attention may allow children who initially had difficulties attending to social-emotional situations to better follow, process, and understand what is happening, thus reducing their anxiety in those social situations. Yet, future research is still required to further explore and deepen our understanding of this topic.

Lastly, regarding prosocial behaviors, our study did not demonstrate any group difference, unlike previous studies (Ketelaar et al., 2015; Tsou et al., 2021). The DHH children in our sample might have a more advanced social development due to several factors. First, thanks to the Chinese governmental support since 2012, a large proportion (73.2%) of DHH children in our sample had bilateral/bimodal hearing through CI and/or HA, which allowed these DHH children living in a predominantly hearing environment to have greater access their daily social world, and in turn, acquire more opportunities for incidental social learning (Broekhof et al., 2021). Second, all DHH children in this study attended intensive rehabilitation programs in a national rehabilitation center. The early intervention/rehabilitation programs of CRRCHSI hold the idea to facilitate both language and social-emotional development of DHH children, by using multiple methods in early interventions: DHH children attend classes with other DHH peers in the center so that they have plenty of opportunities to engage in social interactions; they also receive one-on-one supervision from their teachers hence their mental fitness is safeguarded. Notably, sign language is not the emphasis of education and interventions, while the teachers set up goals to improve children’s listening and speaking skills, cognitive skills, and social adjustment. The parents of these DHH children also attended the programs to learn how to interact or communicate with their children. The relatively high educational levels of the parents in our sample might contribute to stronger support for their children’s social-emotional development effectively (El Nokali et al., 2010). Accordingly, we believe that the intensive care for DHH children can at least partially explain the positive outcomes observed in this study, where DHH children appeared to feel, attend to and react pro-socially to other people’s emotions to a similar extent as their TH peers.

### Limitations and Future Directions

The present study was amongst the first to investigate empathic development among Chinese preschoolers and brought new insights into the role of empathy in preschoolers’ social-emotional development. Nonetheless, several limitations should be noted. First, our study collected data from only two-time points, whereas prior studies showed age-related changes followed children’s development over a longer time (e.g., Li et al., 2023; Tsou et al., 2021). Following children’s development for a longer duration may increase the likelihood of observing developmental changes.

Furthermore, it is notable that 49% of participants dropped out at the second time point, which may increase the likelihood of selection bias in our longitudinal data. Although linear mixed models are known for being good at handling data with attrition (Gustavson et al., 2012), selection biases may still exist and lower the accuracy of estimation in linear regressions. Notably, we found the DHH children who dropped out at the second wave differed from those who stayed in the mean values of internalizing behaviors and social competence, which implies a selection bias that might impact our results. Future research with longer tracking time and lower attrition is needed to verify our findings and to further our knowledge of the empathic development of preschool children.

Second, the present study relied solely on parental report questionnaires. Yet using only one type of measure could result in higher common method bias (Podsakoff et al., 2003). Multi-method designs using other operationalizations of the constructs in the study, such as observational tasks and neuropsychological approaches, can be utilized to improve the validity of the study.

Third, although the socioeconomic status of our participants (e.g., paternal and maternal education level, annual household income) was not different from the national average levels of China (Akimov et al., 2021), caution is still warranted when generalizing our findings to other ethnic/minority groups, as China is a large, populous country with high diversity in population. It is also notable that the preschool children in our sample received intensive early intervention (including auditory training, and special education) in a professional rehabilitation centre. However, on the national level, not all Chinese children have access to such treatment/intervention due to financial or other reasons. The intervention of CRRCHSI might be a reason why we did not detect large group differences between DHH and TH children in their development. In order to improve the external validity of the sample, future studies are recommended to recruit participants from different regions and social classes, so that the overall status of the DHH children in the country can be revealed.

Lastly, the longitudinal design allowed us to observe changes in study variables over time, offering insights into the possible mechanisms and dynamics of development. However, we could not establish whether changes in predicting variables preceded changes in outcomes variables, nor could we rule out reverse causality or bidirectional relationships without additional theoretical and analytical frameworks. Future research that combines the longitudinal approach with experimental manipulations might be of help to unravel the causal links between the study variables.

A practical implication of this study is that timely/early intervention and hearing rehabilitation seem key to children’s social-emotional development over the preschool years. As children’s social-emotional development largely relies on their socialization, researchers and practitioners should explore more effective strategies that mitigate difficulties DHH children encounter in social settings. For example, one-on-one interactions between practitioners and DHH children seems crucial for providing sufficient verbal input for DHH children, which in turn seems to support their early language and social-emotional development. Creating inclusive living and learning environments might be another efficient way to increase children’s daily experiences in social participation, and to establish interpersonal bonds between DHH children and their (often hearing) peers. Furthermore, new techniques such as wearable sensors can be utilized to evaluate the interacting patterns between DHH children and their peers. The data collected by wearable sensors can provide detailed information on DHH preschoolers’ physiological activities (e.g., heart rate, arousal levels), peer relations (e.g., frequencies of interactions), and cognitive abilities (e.g., emotion recognition, reactional tendency), which may be of great help in evaluating DHH children’s social-emotional development and providing well-directed support to promote children’s developmental progress (Pal et al., 2021; Sousa et al., 2023). These suggestions call for verification from future studies to deepen our understanding about how to support the social-emotional growth of DHH children.

## Conclusion

The present study supports previous research stressing the important role that empathy has in preschool children’s social-emotional functioning. However, it also became evident that empathy cannot be studied as a unidimensional concept. For DHH and TH children alike, higher levels of emotion contagion, feeling what the distressed person feels, thus perhaps being more self-focused, seemed quite maladaptive to their psycho-social functioning, relating to more internalizing and externalizing symptoms over time. On the other hand, attention to another person’ s feelings and trying to comfort the other person, which are more other-focused, seemed to show an opposite effect; these aspects of empathy related to better psychosocial functioning instead, over time, and in both groups. These findings seem to align with values commonly taught to children in a collectivistic-oriented country such as China, which may encourage children to be more interdependent and responsive to each other; although it is important to note that the maladaptive function of emotion contagion is also shown in studies with Western children (Tsou et al., 2021). These findings bear clinical importance, as professionals working with these young children should thus also develop a more nuanced understanding of the different aspects that empathy consists of; and being overwhelmed by other people’s emotional responses might denote an inability to self-regulate, but could also imply a high-sensitivity for interpersonal stimuli.

## Data Availability

The data that support the findings of this research are openly available in DataverseNL (https://dataverse.nl/) at 10.34894/VT23CY.
